# Development and Evaluation of Oleanolic Acid Dosage Forms and Its Derivatives

**DOI:** 10.1155/2020/1308749

**Published:** 2020-11-25

**Authors:** Anjie Feng, Shanjing Yang, Yue Sun, Li Zhang, Fumin Bo, Lingjun Li

**Affiliations:** School of Pharmacy, Shandong University of Traditional Chinese Medicine, Jinan 250355, China

## Abstract

Oleanolic acid is a pentacyclic triterpenoid compound that exists widely in medicinal herbs and other plants. Because of the extensive pharmacological activity, oleanolic acid has attracted more and more attention. However, the structural characteristics of oleanolic acid prevent it from being directly made into new drugs, which limits the application of oleanolic acid. Through the application of modern preparation techniques and methods, different oleanolic acid dosage forms and derivatives have been designed and synthesized. These techniques can improve the water solubility and bioavailability of oleanolic acid and lay a foundation for the new drug development. In this review, the recent progress in understanding the oleanolic acid dosage forms and its derivatives are discussed. Furthermore, these products were evaluated comprehensively from the perspective of characterization and pharmacokinetics, and this work may provide ideas and references for the development of oleanolic acid preparations.

## 1. Introduction

**Figure 1:**
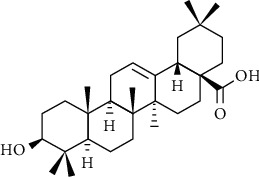
Chemical structures of oleanolic acid.

Oleanolic acid (OA, Figure 1) belongs to oleanane type pentacyclic triterpenes, widely distributed in nearly 200 species of plants, including Swertia, Ligustrum lucidum, grape, and elderberry [1]. Many studies have shown that oleanolic acid has the pharmacological effects of liver protection, antioxidation, hypolipidemia, antitumor, anti-inflammatory, and antiviral [2–6]. However, due to its poor water solubility and low bioavailability of oral administration, the oleanolic acid development in the pharmaceutical field has been limited, and its therapeutic effect is also difficult to be fully exerted [7]. In this paper, by reviewing the preparation technology of oleanolic acid at home and abroad in recent years, it is found that there are mainly two ways to improve the water solubility, permeability, and bioavailability of oleanolic acid. One is to prepare new dosage forms of oleanolic acid, such as nanoparticles, liposomes, solid dispersions, and phospholipid complexes to improve oleanolic acid dissolution, penetration and absorption [8]. The other is to modify the molecular formula of oleanolic acid to obtain more bioactive and extensive derivatives, which are the base for the development of new drugs [9].

In this paper, we summarized and evaluated different oleanolic acid dosage forms and derivatives. Evaluation indicators mainly include solubility, bioavailability, cytotoxicity, and biological half-life. The purpose of this study is to provide reference for the oleanolic acid preparation research and follow-up research.

## 2. Pharmacological Effects of OA and Its Derivatives

### 2.1. Hepatoprotective Effect

The hepatoprotective effect of oleanolic acid makes it useful as an over-the-counter oral drug in China to treat humans for liver disorders. Not only has an oleanolic acid protective effect on acute chemical liver injury but also it has a protective effect on liver fibrosis and cirrhosis caused by chronic liver diseases [10]. Its mechanism may be related to an array of gene expression changes, including the Nrf2-, MT-related genes, and transcription factor farne-soid x receptor (FXR) [11, 12]. Oleanolic acid can cause Nrf2-dependent gene induction by promoting Nrf2 nuclear accumulation, which helps protect the liver from hepatotoxicity induced by acetaminophen [13]. In later studies, it has been shown that the increased nuclear accumulation of Nrf2 by oleanolic acid is due to the activation of Akt and ERK pathways [14]. OA also has protective effect on CCl4-induced liver injury in mice. Jeong suggested that OA inhibited the cytochrome P4502E1 expression [15]. However, Yim et al. [16] believed that the hepatoprotective effects are achieved by enhancing the ability of hepatic glutathione regeneration. In addition, Yu et al. [17] designed and synthesized two kinds of OA prodrugs that have obvious protective effect on CCl4-induced liver injury in mice, and the prodrugs improved the safety and efficacy of the parent drug. What is more, some derivatives of OA enhanced the hepatoprotective effects, such as nanosuspensions [1] and oleanolic acid-amino acids derivatives [18].

### 2.2. Antitumor Activity

OA is one of the most abundant triterpenoids in plants and possesses antitumor activity in many cancer lines, such as liver cancer [19], thyroid cancer [20], lung cancer [21], cervical cancer [22], and gastric cancer [23]. At present, there are many reports on the treatment of liver cancer with OA. Studies have shown that the inhibition of cancer cell growth by OA is mediated via suppression of cancer cell migration and invasion, mitochondrial apoptosis, G2/M cell cycle arrest, and deactivation of JNK/p38 signaling pathway [24, 25]. Yao et al. [26] have proved that OA significantly induced the expression of UGT1As in HepG2, and the induction on UGT1A1 is mediated by PXR activation, not CAR. In addition, studies have found that the OA and autophagy inhibitors combination or the p-glycoprotein inhibition can play a better role in antiliver cancer effect [27, 28]. OA has a large molecular weight and low solubility, which results in low anticancer efficiency. Therefore, researchers have designed a variety of OA derivatives, such as OA/hederagenin-nitric oxide donor hybrid [29], 2-cyano-3, 12-dioxooleana-1, 9(11)-dien-28-oic acid (CDDO), and its C-28 modified derivative: methyl-ester (CDDO-Me) [30], and a gold (I) complex containing an OA derivative [31]. Fan et al. [32] demonstrated that OA covered with nanoliposomes led to enhanced anticancer effects by suppressing proliferation, migration, and invasion. In recent antitumor studies, combination therapy has become a hot spot. Some researchers have prepared calcium carbonate (CC) hybrid nanoparticles (HN) decorated with cancer cell membranes (CM) for the delivery of cisplatin (CDDP) and OA. The results showed that compared to free drugs alone or mono systems, it could enhance cell apoptosis and can reverse the cancer cells multidrug resistance [33, 34].

### 2.3. Anti-Inflammatory Activity

OA has a significant anti-inflammatory effect and is effective against a variety of inflammations [35], such as vasculitis [36], enteritis [37], and tracheitis [38]. Particularly, OA has an inhibitory effect on rat complement and has anti-inflammatory activity in both adjuvant-induced arthritis and carrageenan-induced paw edema in rats [39]. Kim et al. [40] suggested that the anti-inflammatory and antiasthmatic effects of OA may be exerted through inhibition of the GATA-3 and retinoic acid-related orphan receptor *γ* t pathways. In a later study, it was found that the OA anti-inflammatory activity is related to MAPK signaling pathways [41]. Wang et al. [42] demonstrated that OA treatments could dose-dependently ameliorate spinal cord damage through impeding p38- and JNK-regulated apoptosis and inflammation. Therefore, OA can be served as an effective therapeutic agent for spinal cord injury treatment. Furthermore, in vitro biological tests indicated that some OA derivatives showed significant anti-inflammatory activities, which suggested that hydroxylation at C-7 and glycosylation at C-28 had benefit effects [43].

### 2.4. Fight Metabolic Syndrome

Metabolic syndrome is composed of abnormal metabolism factors such as dyslipidemia, impaired glucose tolerance, insulin resistance, inflammation, obesity, and hypertension. Typically, metabolic syndrome is associated with many pathologies, especially type 2 diabetes mellitus, obesity, and cardiovascular diseases [44–47]. OA has potential protective effects on short-term and long-term metabolic dysfunction fructose-induced in rats [48]. And its mechanism of action may be related to hepatocyte nuclear factor 1b, an important regulator of glucose and lipid metabolism [49]. Many OA derivatives have therapeutic effects on metabolic diseases, such as patent CN 103739652A (*α*-glucosidase inhibitors), CN102838651A (OA derivatives as hypoglycemic agents), and CN1682740A (OA derivatives as glycogen phosphorylase inhibitors) [50].

The obesity prevalence has been increasing alarmingly, and it has become a global concern. The OA antiobesity effects have been reported in mice [51–53]. Kim et al. [54] showed that OA inhibits the inflammatory response during adipocyte differentiation through blocking IL-6-TRAF6-NF-*κ*B signaling and visfatin, a proinflammatory and visceral fat-specific adipokine expressed in adipocytes [55]. OA can inhibit fat production while maintaining total estrogen levels, which may be related to the significant downregulation of ACC, a key gene for fat synthesis [56]. In addition, obesity leads to chronic inflammation in the whole body, yet SO1989 (a derivative of OA) can restore the balance between M1 polarized and M2 polarized macrophages in obese mice induced by high fat diet (HFD), thereby improving fat inflammation and metabolic dysfunction [57]. Recent study has shown that the administration of OA to prediabetic rats can improve body/liver weight ratio, and significantly reduced plasma triglycerides and very low-density lipoproteins [58]. DKS26 (a derivative of OA) plays the role of hypoglycemic, hypolipidemic, and islets protective effects through cAMP/PKA signaling pathway [59]. Moreover, nano-OA can effectively improve the metabolic dysfunction induced by high-fat candy diet in rats by improving its bioavailability and pharmacodynamic properties [60]. In conclusion, OA and its derivatives are potential drugs against metabolic syndrome.

### 2.5. Other Pharmacological Activities

OA has the antioxidation effect, and its mechanism may be related to the antioxidant's generation increase and the expression of oxidative stress-sensitive transcription factors-Nrf2 and MAP kinases [61, 62]. Studies have reported that OA exerts neuroprotective effects by inhibiting the activated oxidative stress and inflammatory response of microglia cells associated with Alzheimer's disease [63–65]. In addition, OA also has antiosteoporosis, antiproliferation of prostate cells, antimuscle atrophy, anti-influenza, and antidepression effects [66–70].

## 3. New Dosage Form of Oleanolic Acid

The pharmacokinetic data of different dosage forms of oleanolic acid are shown in Table 1. As is shown in Table 1, the method used in most of the experiments is LC-MC, the experimental object is SD rats, and the absolute bioavailability and relative bioavailability are given. It can be seen that the new dosage forms have improved to varying degrees in bioavailability compared to commercially available oleanolic acid tablets. Next, we will conduct a specific analysis of the new dosage forms.

### 3.1. Cyclodextrin Inclusion

Through its external hydrophilic and internal hydrophobic structure, cyclodextrin can wrap many small molecular drugs to improve the water solubility of insoluble drugs [78]. In fact, different types of cyclodextrins have different intensities of increasing water solubility. Li et al. [79] prepared oleanolic acid *β*-cyclodextrin inclusion compound by saturated aqueous solution method and measured the drug solubility by HPLC. The result showed that the inclusion compound solubility in water increased by 3.4 times. After adding NaOH to form a salt, solubility increased by 107 times. While the polyvinylpyrrolidone addition cannot improve the saturates solubility and also affect the oleanolic acid activity. Wang [72] used hydroxypropyl *β*-cyclodextrin to prepare oleanolic acid inclusion compound and established a pharmacokinetic detection method (HPLC-MC) after oral administration of oleanolic acid. Finally, the absolute bioavailability of acid-hydroxypropyl *β*-cyclodextrin inclusion compound in rats was analyzed, and the results showed that the absolute bioavailability was 2.010%, compared with relevant literature reports [80]; oral bioavailability (*F* = 0.7%) has been improved to a certain extent. Ren et al. [80] used synthetic long-chain amino *β*-cyclodextrin derivatives tetra-ethylene pentamine-*β*-cyclodextrin to prepare oleanolic acid inclusion compounds. It can be concluded that the oleanolic acid water solubility was improved 2100 times. Oprean et al. [81] chose the antitumor activity in vitro of oleanolic acid cyclodextrin inclusion compound as the index and selected 2-hydroxypropyl-b-cyclodextrin as the most suitable inclusion material. Conversely, it is reported that [82] the interaction strength between oleanolic acid and natural *β*-cyclodextrin derivatives (*β*-CDs) is higher than that of the corresponding hydroxypropyl modified *β*-CDs, and the complexation constant of *γ*-CDs is 6 times of *β*-CDs. The oleanolic acid preparation by cyclodextrin inclusion technique can indeed improve the solubility, but the research on its metabolic absorption and bioavailability in vivo is still insufficient.

### 3.2. Solid Dispersion

Solid dispersion (SD) preparation process is relatively simple, which is one of the means to improve the dissolution and bioavailability of drugs. Commonly used carriers for solid dispersions include povidone (pvp) [83], cross-linked povidone (pvpp) [73], and nano calcium carbonate [84]. Some researchers chose PVPk30-Soluplus composite carrier to prepare oleanolic acid solid dispersion, which can obviously increase the dissolve and dissolution rate of this component and improve its dissolution effect [85]. Gao et al. [83] developed the amorphous solid dispersions of OA (OA-PVP) by the hot-melt extrusion, which avoided the organic solvent use, so it was safe and green [86]. The evaluation in vitro and in vivo showed a remarkable improvement of OA-PVP compared with commercial tablet. Especially, pharmacokinetic analysis in rats showed that the bioavailability of OA-PVP was about 2.4 times that of OA tablets. As for instance, Wang et al. [73] designed oleanolic acid-polyvinylpolypyrrolidone (OA-PVPP-SD) by a simple solvent evaporation method. Dissolution test showed that approximately 50%-75% of OA was dissolved from SDs within the first 10 min, which is about 10-15 times of free OA. In vivo pharmacokinetic studies showed that OA-PVPP-SD has a shorter *T*_max_ (*P* < 0.05) and an increased *C*_max_ (*P* < 0.05) compared to direct oral administration of OA, and its relative bioavailability is 183.07%. In summary, OA-SD made of composite carrier can significantly improve the OA dissolution rate in vitro, but the bioavailability needs further study.

### 3.3. Nanometer Preparation

Nanometer preparations have become a research hotspot in recent years, and nanotechnology has unique advantages in improving the solubility, slow and controlled release, and targeting of poorly soluble drugs [87]. In recent years, many researchers have applied nanotechnology to oleanolic acid preparation. Next, various nanometer preparations will be discussed in detail.

#### 3.3.1. Self-Microemulsifying Drug Delivery System

Yang et al. [88–90] conducted a more comprehensive study on the self-microemulsifying drug delivery system of oleanolic acid (SMEDDS-OA). The results of SMEDDS-OA dissolution test in vitro showed that the cumulative dissolution rate of the drug in 15 minutes reached more than 85%, while the reference marketed tablets were less than 60%, and the solubility increased by more than 1000 times compared with OA [88]. Through in vivo pharmacokinetic studies in rats, SMEDDS-OA has an earlier *T*_max_ and a higher *C*_max_ than the reference tablet, and its relative bioavailability has reached 507.03% [89]. Xie et al. [74] also studied the pharmacokinetics of SMEDDS-OA in rats. And likewise compared with oral tablets, its relative bioavailability reached 402.24%. Those suggest that self-microemulsifying drug delivery system can improve the solubility and bioavailability of OA. In a word, the basic experimental research is more comprehensive, but there are few reports about clinical trials.

#### 3.3.2. Nanoparticles

**Figure 2:**
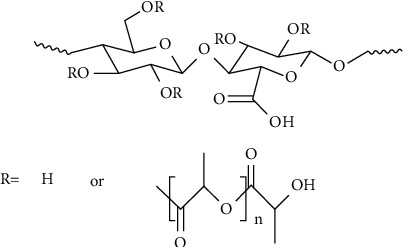
Amphiphilic carboxylated cellulose-graft-poly (CC-g-PLLA).

By preparing OA nanoparticles, OA can achieve the purpose of slow release and targeting, so as to better play the antitumor effect [91, 92], improve the drug safe, and expand the medication scope [75, 93]. Xia et al. [93] developed a novel OA nanoparticle (OA-NP)-loaded lactoferrin nanodelivery system. The dissolution experiment in vitro showed that at 20 minutes, the dissolution rate of OA-NPS was 80%, and that of the control OA was only 40%. The pharmacokinetic study in rats showed that its relative bioavailability was 320.5%. Wang et al. [94] encapsulated OA into nanoparticles with amphiphilic carboxylated cellulose-graft-poly (CC-g-PLLA, Figure 2), which can improve water solubility and release time of the drug and show strong antitumor activity. Khan et al. [95] developed a pH-dependent calcium carbonate nanodelivery system for the combination of chemotherapeutic drugs cisplatin and OA in the hepatocellular carcinoma treatment. Research showed that it can mitigate the liver toxicity caused by chemotherapy drugs and overcome drug resistance. Moreover, some researchers [96, 97] prepared nanoparticles with TPGS-PLGA and mPEG-PLGA-loaded OA, respectively, which can improve the antibacterial and antitumor activities. In vitro study showed that TPGS-stabilized OA-loaded PLGA nanoparticles were more sensitive to pathogenic bacteria. For example, TPGS-PLGA-OA is 25-fold more selective than pure OA in case of wild type strain. Similarly, mPEG-PLGA-OA exhibits stronger cytotoxicity to cancer cells and is more effective for the delivery of OA.

#### 3.3.3. Nanoliposome

Liposomes have attracted people's attention because of their similar structure to biofilm structure, strong drug loading, and transport capacity. Developing oleanolic acid into nanoliposomes has slow and controlled release, targeting, reducing toxic and side effects, and enhancing the antitumor effects [98]. In addition, the preparation of nanoliposomes of Adriamycin and OA can reduce the Adriamycin cardiotoxicity but does not affect the anticancer activity [99]. Liu et al. [76] prepared hydrophilic polyvinylpyrrolidone K30 (PVP-K30) modified OA liposomes. The results of the characterization studies showed that the average particle size was 179.4 nm, the encapsulation efficiency was above 90%, and the oral bioavailability reached 607.9%. Li et al. [100] designed and prepared a novel octreotide-modified magnetic liposomes (OMlips) for magnetically-orienting and receptor-mediated dual-targeting anticancer drug delivery. Using OA as a drug, OA-OMLips was prepared. In vitro drug-release study, about 23% of OA was released from OA-OMLips within 12 hours, which delayed the drug release. The antitumor effect studies in vivo showed that the tumor volume of OA-OMLips with magnetic group is 53% smaller than that of OA-OMLips without magnetic group, indicating that the drug delivery system effectively increases the drug accumulation in tumor tissues and improves antitumor effect. In summary, the OA antitumor activity makes it often used as an adjuvant for the development of combination therapies for cancer.

#### 3.3.4. Micelle

Micelles can improve the solubility, dissolution, and bioavailability of OA. Commonly used preparation methods include dialysis, solvent evaporation, and film dispersion [101]. For instance, Hao [5] prepared OA-loaded mixed micelles using ethanol thin-film hydration method, characterized OA micelles, and studied in vitro release and in vivo drug efficacy. The results showed that the average particle size of OA micelles in the aqueous phase was 95.7 ± 3.6 nm, DL is 3.5%, and EE is 93.6 ± 0.05%. The drug release studies in vitro showed that about 80% of OA released from the dialysis bag containing free OA at 24 h, while OA micelles are only 40%, which achieves the purpose of sustained release. Furthermore, OA-micelles displayed higher antitumor efficacy than free OA in both A549 and PC-9 cells. The results showed that OA micelles can significantly reduce tumor size, inhibit tumor invasion, migration, and epithelial-mesenchymal transition in vivo. In conclusion, polymeric micelles are a promising nanodrug delivery system for lung cancer.

#### 3.3.5. Nanometer Emulsion

Some researchers [102] prepared OA-loaded nanoemulsions with an average particle size of less than 60 nm. Stability test studies showed that OA nanoemulsions have good physical properties (small droplet size, low viscosity), stable for at least 1 month, without instability. The results of experiments in vivo showed that the preparation is nontoxic and nonirritating to the skin, and the nanometer emulsion has a high ability to penetrate the skin, which can enhance the OA anti-inflammatory effect. Nanoemulsion made of OA can play a better role in anti-inflammatory after percutaneous absorption.

#### 3.3.6. Phospholipid Complex

Jan et al. [77] prepared a solidified phospholipid complex (OPCH) consisting of OA-phospholipid complex (OPC) and hydroxyapatite (HA) using a simple solvent evaporation method. Studies showed that the water solubility of OPCH is 15.3 times higher than that of OA, and the cumulative dissolution rate of OPCH in vitro is 2.23 times higher than that of OA at 2 h. Furthermore, the combination of OPCH and ketoconazole can inhibit the OA metabolism in the intestinal tract and improve the OPCH bioavailability, which is 2.72 times higher than that of OA. In conclusion, OPCH is designed to improve intestinal absorption of OA, and its OA water solubility and bioavailability are improved in different degrees.

#### 3.3.7. Multivesicular Liposomes

Some researchers [103, 104] prepared OA-encapsulated multivesicular liposomes (OA-MVLs) by a double-emulsion method and characterized them. The results showed that the average particle size of OA-MVLs was 11.57 *μ*m, the encapsulation efficiency of 82.3% ± 0.61%, zeta absolute potential was -13.35 mv, and polydispersity index was 0.21. In vitro studies showed that OA-MVLs can be better absorbed by HepG2 and enhance the growth inhibitory effect on HepG2 cells. Further in vivo experiments on H22 tumor-bearing mice showed that OA-MVLs have better antitumor effects than OA solution. Especially, OAMVLs had a significant inhibitory effect on the liver cancer cells survival at 160 *μ*mol/L but had no significant effect on the normal liver cells viability, which proved that the drug is safe for clinical application.

## 4. Derivatives of Oleanolic Acid

**Figure 3:**
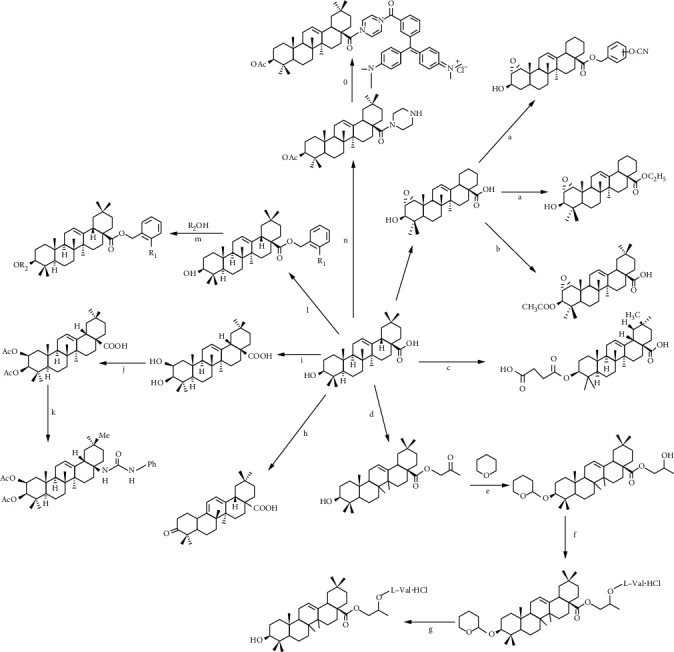
Synthesis of derivatives of oleanolic acid.

The main structural modification sites of oleanolic acid are A ring, c-28, and C ring. The derivatives modified at different positions have different pharmacological activities, and their structure-activity relationships are shown in Table 2. The synthetic route of the main derivatives is shown in Figure 3.

Reagent and conditions are as follows: (a) K2CO3, DMF, substituted benzyl bromide or haloalkane, rt; (b) (1) NaH, DMF, CH3I, rt; (2) 5% NaOH, DMF, rt; (3) acid anhydrides, DMAP, THF or acid, DMAP, EDCI, DCM; (c) succinic anhydride, DMAP, CH2Cl2, rt, 24 h, 87–97%; (d) ClCH2COCH3, DMF, K2CO3, 60°C; (e) CH2Cl2, rt; (f) (1) NaBH4, CH3OH, 0°C; (2) Boc-amino acid, DCC, DMAP, CH2Cl2, rt; (g) (1) p-CH3C6H4SO3H, CH3OH, rt; (2) (C2H5)2O, dry HCl gas, 0°C, then NaHCO3aqueous solution; (h) chromic acid solution, acetone, 0°C, 1 h [114]; (i) (1) SiO2, Jonesreagent, acetone, 30 min, 0°C, 98%; (2) pyridiniumtribromide, AcOH, 2 h, 25°C, quant; (3) NaOH, DMF, inert gas, 0°C, quant; (4) NaBH4, MeOH, THF, 0°C, 1 h, 48% and 45% [115]; (j) Ac2O, TEA, DMAP, DCM, 25°C, 20 h, 76-78%; (k) (1) Diphenylphosphoryl azide, TEA, toluene, 180°C, 3 h, microwaves, 76%; (2) HClaq, THF, 30-50°C, 2-24 h, 76%; (3) phenylisocyanate, toluene, 2 h, 25°C, 69%; (l) DMF, K2CO3, room temp; (m) EDCI, DMAP, reflux, room temp; (n) (1) Ac2O, NEt3, DMAP, DCM, 88%; (2) oxalyl chloride, THF, DCM, piperazine 49%; (0) oxalyl chloride, THF; NEt3, DMAP,25°C, 2 h, 50%.

### 4.1. Structural Modification of a Ring

**Figure 4:**
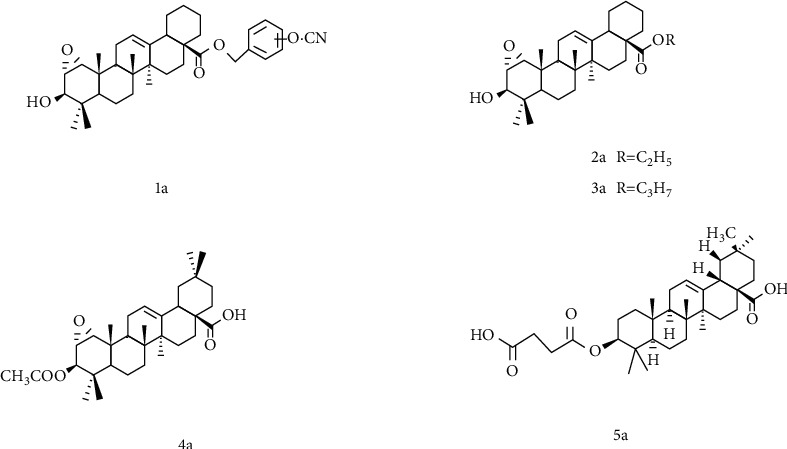
Chemical structure of oleanolic acid derivatives 1-5a.

Liang et al. [105] designed and synthesized a series of 1*α*, 2*α*-epoxy-3*β*-hydroxy oleanolic acid derivatives. The antibacterial activity in vitro was studied, and the results showed that 1a (Figure 4) had good antibacterial activity against Escherichia coli and Bacillus subtilis, and in vitro inhibition rate could reach 60% and 80%, respectively, which is equivalent to streptomycin. 2a and 3a had better inhibitory activity on Klebsiella pneumonia, similar to clindamycin. 4a had better inhibitory activity on the growth of Baumann bacilli. In vitro antibacterial activity studies indicated that the introduction of an ortho-cyano-substituted benzyl group and a short-chain alkyl ester at the 28-carboxyl might improve the antibacterial activity of 1*α*, 2*α*-epoxy-3*β*-hydroxy oleanolic acid. And introducing an acetyl group at the 3-hydroxyl group may improve the OA derivatives inhibitory activity against bacteria. As described, these newly designed OA derivatives have unique antibacterial activity and may be potential antibacterial drugs in the future.

In addition, there is a report [106] that for oleanolic acid derivatives, the role of free carboxylic acid makes it have better antiparasitic activity. Zou et al. [107] analyzed the structure-activity relationship (SAR) of a series of semisynthetic OA derivatives. The results demonstrated that the inhibition of human carboxylesterase 1 (hCE1) by the 28-carboxyl group of OAs is very essential, and the OA modifications at this site including esters, amides, and alcohols are unbeneficial for hCE1 inhibition. In contrast, the modifications of the C-3 hydroxyl group are beneficial to the inhibition of hCE1. Substituting ketones or esters for the C-3 hydroxyl group can increase the inhibition effect of hCE1 and have a high selectivity to hCE2. Among them, the C-3 hydroxyl group is converted into 3-O-*β*-carboxypropionyl group, the inhibitory effect of compound 5a on hCE1 is significantly enhanced, and the selectivity to hCE2 is also very high, which can be used as a drug candidate for the development and design of hCE1. It can be seen from the above that the retention of the C-28 carboxyl group is beneficial to exert certain biological activities of oleanolic acid.

### 4.2. Structural Modification at C-28

**Figure 5:**
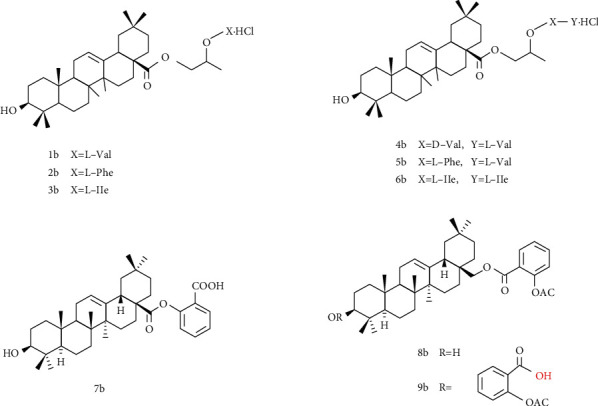
Chemical structure of oleanolic acid derivatives 1-9b.

Cao et al. [116] designed and synthesized seven propylene glycol-linked amino acid diester prodrugs of OA, which were designed to target peptide transporter 1 (PepT1). The water solubility of compound 1-6b (Figure 5) is 35.3, 23.4, 12.3, 69.6, 58.2, and 83.2 *μ*g/mL, respectively, which is significantly higher than that of OA with poor water solubility. In situ rat single-pass intestinal perfusion model and Caco-2 cell model were used to screen the effective permeability and affinity of the prodrug to PepT1. The results showed that compound 1b and 5b were superior to other compounds. Pharmacokinetic experiments were carried out after oral administration of 1b and 5b. The results demonstrated that compared with OA group, *C*_max_ and AUC_0−24_ increased 3.04-fold (*P* < 0.01) and 3.55-fold (*P* < 0.01) in 1b group, *C*_max_ and AUC_0−24_ increased 2.62-fold (*P* < 0.01) and 3.39-fold (*P* < 0.01) in 5b group, which increased the oral bioavailability of OA. In conclusion, the design of oleanolic acid prodrugs targeting peptide transporter 1 is an effective way to overcome the poor solubility of OA and the low oral availability.

Ji's group linked aspirin to the carboxyl group at position 28 to obtain a series of conjugates 7-9b [108]. The inhibitory activity of oleanolic acid-aspirin conjugate on 5-hydroxy tryptamine synthesis and the activity of promoting bone formation were investigated. Among them, the conjugate 7b had the strongest inhibitory effect on serotonin, with the inhibition rate as high as 87.2%, slightly higher than that of the positive control LP533401, which was 12 times that of oleanolic acid and 14 times that of aspirin. In the experiment of promoting osteoblast activity, conjugate 7b also showed the strongest activity, which was close to the proliferation effect of the positive control drug. By comparing conjugate 8b and 9b with 7b, it can be concluded that the carbonyl group at position 28 of OA is a pharmacophore for antiserotonin synthesis, and the modification of hydroxyl at position 3 has little effect on the activity.

### 4.3. Structural Modification of C Ring

**Figure 6:**
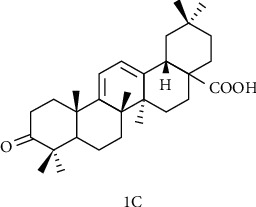
Chemical structure of oleanolic acid derivatives 1C.

The OA derivative 1C (Figure 6) has hepatoprotective effects, and it is superior to lamivudine in suppressing the rebound of the viral replication rate. Some researchers have studied the protective effect of 1C on liver injury induced by carbon tetrachloride (CCl4) in rats and comprehensively evaluated the 1C liver protective effect using histological assay, immunohistochemical staining, and acute toxicity tests [117]. Histological assay showed that the 1C high-dose group showed obvious relief in abnormal areas, and liver cells returned to a healthy state. Nevertheless, the low-dose group also had a moderate degree of relief. Moreover, 1C can inhibit the expression of cytokine TGF-*β*1, which is closely related to liver cell fibrosis, and exert its antiliver fibrosis effect. And the acute toxicity test showed that 1C has low toxicity. Single-dose oral 1C pharmacokinetic studies showed that *C*_max_ was 8.18 ± 0.66 *μ*g/mL, AUC_0−24_ was 90.21 ± 15.68 *μ*g h/mL, and T_½_ was 2.19 ± 0.7 h, eliminating half-life longer than OA, suggesting 1C is potential drugs against liver fibrosis.

### 4.4. Structural Modification of A Ring and C-28 Position

#### 4.4.1. A Ring Modification and the Amidation of C-28

**Figure 7:**
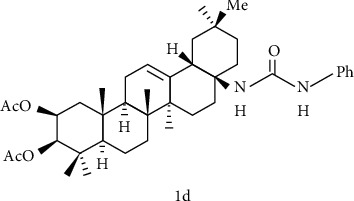
Chemical structure of oleanolic acid derivatives 1d.

Sommerwerk et al. [109] synthesized maslinic acid with oleanolic acid, modified with A ring as well as amidated with C-28 to synthesize a series of OA derivatives to study their toxicity and selectivity to human tumor cells. The results showed that the EC_50_ of compound 1d (Figure 7) to A2780 ovarian cancer cells was 0.9 *μΜ*, and to fibroblasts EC_50_ >120 *μΜ*, the effect was the best. This indicates that the introduction of acetyl groups at the C-2 and C-3 positions, and the presence of (2*β*, 3*β*)-configurated centers, as well as a phenylurea moiety at C-28 are very necessary for obtaining high cytotoxicity and retaining the selectivity between malignant cells and mouse fibroblasts.

**Figure 8:**
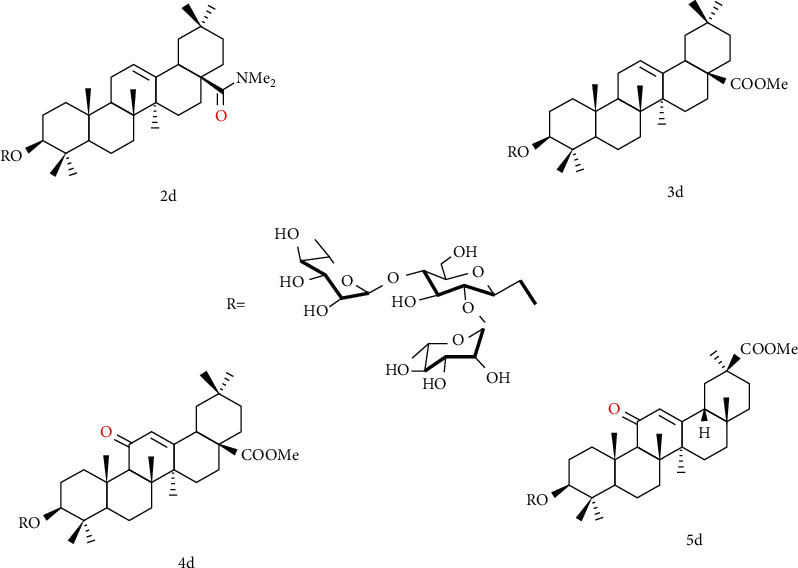
Chemical structure of oleanolic acid derivatives 2-5d.

Song et al. [118] designed and synthesized a series of oleanolic acid derivatives and evaluated them as avian influenza virus H5N1 entry inhibitors. Structure-activity relationship (SARs) studies have shown that fine modification with OA as an aglycon has an important effect on antiviral activity. Whether introducing a disubstituted amide structure at C-28 of OA or changing the C-3 configuration of OA from 3*β* to 3*α*, the OA selectivity index can be significantly improved while maintaining its antiviral activity in vitro. Among these derivatives, compound 2d (Figure 8) showed excellent anti-H5N1 activity in the inhibitory activity experiment, with IC_50_ = 2.36 *μ*M lower than the positive control CL-385319 (IC_50_ = 4.45 *μ*M), which can be used as a lead for further development of H5N1 entry inhibitors compound or scaffold. What is more, the inhibitory activity of compounds 3d, 4d, and 5d (Figure 8) on H5N1 is similar, indicating that the diversity of pentacyclic triterpene structural framework has no significant effect on its anti-H5N1 activity.

**Figure 9:**
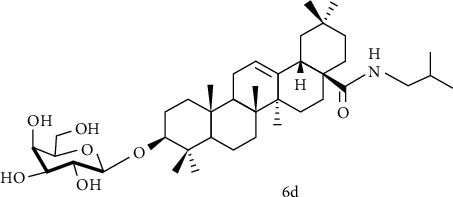
Chemical structure of oleanolic acid derivatives 6d.

Yu et al. [110] used oleanolic acid 3-O-*β*-D-glucuronopyranoside (OAGP) as the raw material, after amidation at the C-28 position and modification of the sugar group, the resulting compound 6d (Figure 9). Pretreatment of H9c2 cells with compound 6d exhibited a better protective effect than OAGP. The survival rate increased from 49.69% (with H_2_O_2_ treatment alone) to 58.69%, but the effect is not as good as derivatives of ursolic acid skeleton. In summary, the experimental results showed that the amidation of C-28 and the connection of isobutyl are beneficial for the cardiomyocytes protection.

#### 4.4.2. A Ring Modification and C-28 Esterification

**Figure 10:**
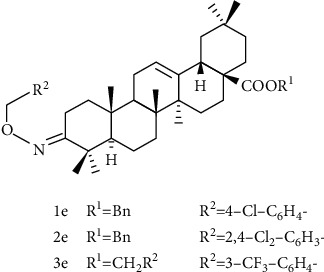
Chemical structure of oleanolic acid derivatives 1-3e.

Hu et al. [111] used oleanolic acid as the leader, introduced oxime ether structure at C-3 position, introduced methyl group and benzyl group, and substituted aromatic hydrocarbon at C-28 carboxyl position to obtain compound 1-3e (Figure 10). Compounds 1-3e all showed a certain inhibitory activity on glucosamine-6-phosphate synthase (GlmS), but the effect was comparable to OA. Moreover, compounds 1e and 2e showed better bactericidal activity against Sclerotinia sclerotiorum, Botrytis cinerea of tomato, and rice blast, which was significantly better than OA, but lower than that of positive control drugs. From this report, it can be seen that OA introduces an oxime ether structure, and esterification at the C-28 position does not significantly improve anti-Glms activity.

**Figure 11:**
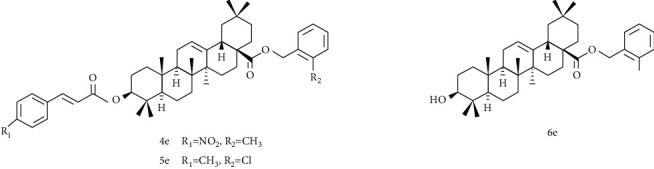
Chemical structure of oleanolic acid derivatives 4-6e.

In recent years, it has become a hot spot to modify the A ring of OA and esterify the C-28 position to connect various pharmacophore to study its antitumor activity [119, 120]. Wang et al. [112] designed and synthesized a series of OA-cinnamate derivatives using molecular hybridization approach. And the MTT method was used to determine the cytotoxicity of cervical cancer HeLa, breast cancer MCF-7, and normal liver cells L-O2 in vitro. The results showed that 4e (Figure 11) had the strongest selective killing effect on HeLa cells, IC_50_ = 1.35 *μ*M, and 5e (Figure 10) on MCF-7 had the strongest selective killing effect, IC_50_ = 1.79 *μ*M. In addition, compound 6e (Figure 10) also showed strong selective inhibitory activity on HeLa cells, IC_50_ = 1.55 *μ*M. From the above results, we know that the introduction of a benzyl group with a substituent at the C-28 position can enhance its anticancer activity. The 4-methylcinnamic acid linked to the A ring can significantly increase the inhibitory effect of OA on MCF-7 cells.

**Figure 12:**
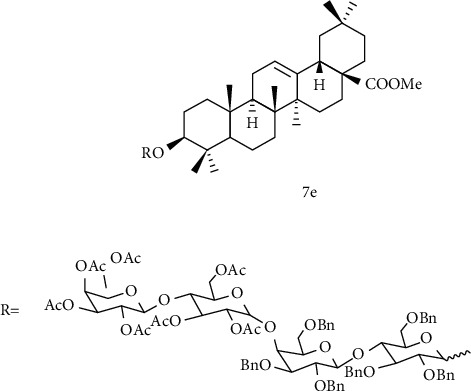
Chemical structure of oleanolic acid derivatives 7e.

Deng et al. [121] used oleanolic acid aglycone as the starting material to esterify the C-28 position and introduce sugar group at C-3 position to obtain the OA derivative 7e (Figure 12). Human colon cancer cells (HCT8) were selected for in vitro antitumor activity experiment, in which the inhibition rate of OA glycoside derivative 7e reached 98.26% at a concentration of 1 × 10^−3^ mmol/L, which was equal to that of the positive control drug Adriamycin, but not as good as Adriamycin at other concentrations. Although the antitumor effect of the derivative 7e is not ideal, it improves the water solubility of the derivatives, suggesting that glycosyl modification can be used to improve the water solubility of insoluble compounds.

#### 4.4.3. A Ring Modification and C-28 Position Connects Nitrogen-Containing Heterocycle

**Figure 13:**
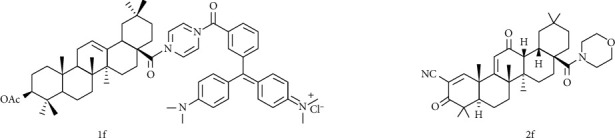
Chemical structure of oleanolic acid derivatives 1f and 2f.

In order to improve the oleanolic acid antitumor activity, some researchers have conducted some beneficial explorations. Friedrich [122] acetylated the C-3 position, and the nitrogen-containing heterocycle at the C-28 position was attached to the scaffold with a cationic functional group to obtain compound 1f (Figure 13). And 1f had good cytotoxicity to human breast cancer cell MCF-7, EC_50_ = 0.7 *μ*M. Its cytotoxicity is 17 times that of the standard substance betulinic acid and 50 times that of OA, which seems to prove that the type of triterpene acid, the type of amide bond, the type of cationic residue, and its substitution pattern have important meaning for tumor cytotoxicity. Gao [113] discussed the potential antitumor activity of oleanolic acid derivative 2f (Figure 13). Using cell viability analysis to calculate IC_50_ for six tumor types, it was found that 2f has a broad antitumor effect and its IC_50_ on osteosarcoma cells significantly lower than other tumor cells, indicating that it tends to inhibit the growth of osteosarcoma cells. In vivo antitumor experiments in nude mice suggested that 2f can reduce tumor volume and weight and has low toxicity. Given the above results, its molecular mechanism is related to the downregulation of the expression of glycolysis-related enzymes in nude mice.

## 5. Conclusion

In conclusion, the new dosage forms preparation and the chemical structure modification of OA can improve the bioavailability and expand the scope of application, which are two effective ways to design and develop new drugs. Although the methods have made great progress, there are still some problems that need further research and exploration. For example, there are few innovative studies, the modified dosage form has not been biologically tested, and the activity of the obtained derivative has been decreased or no significant improvement. In the future research, we should make clear the purpose of the experiment, learn from the predecessors' experience, and study the necessary functional groups, pharmacophore, and unnecessary substituents, so as to carry out accurate and reasonable modification. Moreover, with the increasing drug resistance of some bacteria and viruses, the development of new drugs from natural products has become a research craze, and pentacyclic triterpenes have attracted much attention because of their various pharmacological activities. It is noteworthy that oleanolic acid exists widely in nature has strong biological activity and great research potential. It needs more researchers to explore in order to develop products for the benefit of mankind as soon as possible.

## Figures and Tables

**Table 1:** Pharmacokinetic data of different dosage forms of oleanolic acid.

Form	Dosage	Administration	AUC	C max	T max	T½	Bioavailability	Object	References
OA solution	0.5 mg/kg	Injection	16 mg min/ml			41.9 min	1	Male rat	[7]
	25 mg/kg	Oral gavages	5.9 mg min/ml	74 ng/ml	25 min	46.5 min	0.7%		
Capsule	40 mg	Oral	124.29 ng h/ml	12.1 ng/ml	5.2 h	8.7 h		Adult male	[71]
Cyclodextrin inclusion	1 mL	Injection	4468.85 mg/L/min	103.97 mg/L	2 min	54.69 min	1	SD rat	[72]
		Oral gavages	89.42 mg/L/min	1.52 mg/L	45 min	42.53 min	2%		
Solid dispersion	10 mg/kg	OA Oral gavages SD-OA	82.3 ng h/ml	24.95 ng/mL	1.25 h	4.99 h	183.07%	SD rat	[73]
			150.7 ng h/ml	99.58 ng/mL	0.49 h	2.23 h			
Tablet	50 mg/kg	Oral gavages	25 mg h/ml	201.33 ng/mL	2.92 h	10.08 h		SD rat	[74]
SMEDDS	50 mg/kg	Oral gavages	1749.29 mg h/ml	70.09 ng/mL	2.02 h	4.17 h	402.24%	SD rat	[74]
Nanoparticles	10 mg/kg	Oral gavages	126.53 ng h/ml	12.6 ng/mL	0.33 h		340.5%	SD rat	[75]
Nanoliposome	50 mg/kg	Oral gavages	2471.7 ng h/ml	542.78 ng/mL	0.7 h	1.21 h	607.9%	SD rat	[76]
Phospholipid complex	50 mg/kg	Oral	360.6 ng h/ml	78.7 ng/mL	0.46 h		139.4%	SD rat	[77]

**Table 2:** Structure-activity relationships for the main categories of derivatives.

The position of the modification	Representative derivatives	Activities	References
A ring	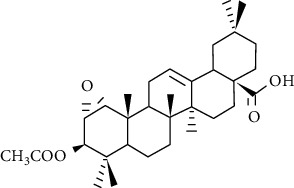	Antibacterial activity	[105]
		Antiparasitic activity	[106]
		Inhibitors against hCE1	[107]
C-28	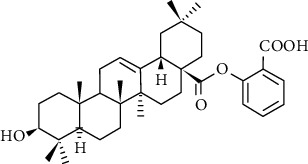	Inhibitory effect on serotonin	[108]
		Promoting osteoblast activity	
C ring	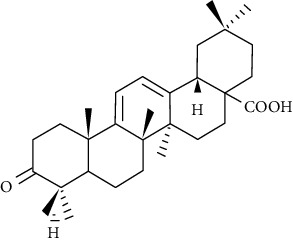	Hepatoprotective effect	[109]
A ring and c-28 (amidation)	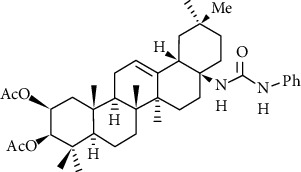	Antitumor activity	[109]
		Protective effect of cardiomyocytes	
			[110]
A ring and c-28 (esterification)	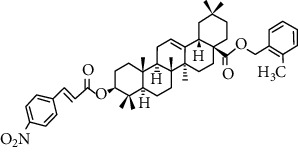	Antimicrobial activity	[111]
		Antitumor activity	[112]
A ring and c-28 (nitrogen-containing heterocycle)	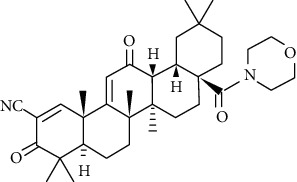	Antitumor activity	[113]

## Data Availability

The data used to support the findings of this study are available from the corresponding author upon request.
